# Effects of Sunitinib and Other Kinase Inhibitors on Cells Harboring a *PDGFRB* Mutation Associated with Infantile Myofibromatosis

**DOI:** 10.3390/ijms19092599

**Published:** 2018-09-01

**Authors:** Martin Sramek, Jakub Neradil, Petra Macigova, Peter Mudry, Kristyna Polaskova, Ondrej Slaby, Hana Noskova, Jaroslav Sterba, Renata Veselska

**Affiliations:** 1 Laboratory of Tumor Biology, Department of Experimental Biology, Faculty of Science, Masaryk University, 61137 Brno, Czech Republic; martin.sramek@mail.muni.cz (M.S.); jneradil@sci.muni.cz (J.N.); macigova@med.muni.cz (P.M.); 2 Department of Pediatric Oncology, University Hospital Brno and Faculty of Medicine, Masaryk University, 66263 Brno, Czech Republic; mudry.peter@fnbrno.cz (P.M.); polaskova.kristyna@fnbrno.cz (K.P.); sterba.jaroslav@fnbrno.cz (J.S.); 3 International Clinical Research Center, St. Anne’s University Hospital, 65691 Brno, Czech Republic; 4 Central European Institute of Technology, Masaryk University, 62500 Brno, Czech Republic; ondrej.slaby@ceitec.muni.cz (O.S.); hana.noskova@ceitec.muni.cz (H.N.)

**Keywords:** infantile myofibromatosis, receptor tyrosine kinases, platelet-derived growth factor receptor, protein kinase inhibitors, sunitinib, erlotinib, FR180204, U0126, targeted therapy

## Abstract

Infantile myofibromatosis represents one of the most common proliferative fibrous tumors of infancy and childhood. More effective treatment is needed for drug-resistant patients, and targeted therapy using specific protein kinase inhibitors could be a promising strategy. To date, several studies have confirmed a connection between the p.R561C mutation in gene encoding platelet-derived growth factor receptor beta (PDGFR-beta) and the development of infantile myofibromatosis. This study aimed to analyze the phosphorylation of important kinases in the NSTS-47 cell line derived from a tumor of a boy with infantile myofibromatosis who harbored the p.R561C mutation in PDGFR-beta. The second aim of this study was to investigate the effects of selected protein kinase inhibitors on cell signaling and the proliferative activity of NSTS-47 cells. We confirmed that this tumor cell line showed very high phosphorylation levels of PDGFR-beta, extracellular signal-regulated kinases (ERK) 1/2 and several other protein kinases. We also observed that PDGFR-beta phosphorylation in tumor cells is reduced by the receptor tyrosine kinase inhibitor sunitinib. In contrast, MAPK/ERK kinases (MEK) 1/2 and ERK1/2 kinases remained constitutively phosphorylated after treatment with sunitinib and other relevant protein kinase inhibitors. Our study showed that sunitinib is a very promising agent that affects the proliferation of tumor cells with a p.R561C mutation in PDGFR-beta.

## 1. Introduction

Infantile myofibromatosis (IM; [MIM#228550]) is a disorder of mesenchymal proliferation characterized by the development of nonmetastatic tumors [1] that present as firm, flesh-colored to purple nodules usually located in the skin, subcutaneous tissues, bone, muscle or visceral organs [2,3]. This disease was described under different names, the name “infantile myofibromatosis” was first used in 1981 [4]. Although rare, with an incidence of 1 in 400,000 children, IM represents the most common proliferative fibrous tumor of infancy [5,6]. Myofibromas are usually present at birth or develop shortly thereafter, and almost 90% of the tumors are diagnosed before the age of two years, with a median age of three months [6,7,8]. A male predominance has been reported, and the ratio of male to female patients varies from 1.5:1 to 1.8:1 [5].

IM clinically presents in three main forms: (1) Solitary, (2) multicentric without visceral involvement, and (3) multicentric with visceral involvement [6]. The prognosis is excellent in solitary or multicentric nonvisceral forms with a possibility of spontaneous regression of the lesions but is poor when detected in the viscera [9]. Surgical excision of a single lesion is the standard of care [8]. Multiple lesions or surgically unresectable lesions are treated using various therapeutics, such as anti-inflammatory drugs, interferon-alpha, vinblastine, vincristine, dactinomycin, cyclophosphamide and methotrexate [6,8].

The molecular pathogenesis of IM is not completely understood. Familial forms exhibiting autosomal dominant and recessive transmission have been reported over the past two decades [10]. In 2013, several point mutations in the platelet-derived growth factor receptor beta (PDGFR-beta) gene (*PDGFRB*) were identified to be associated with familial IM. A study of nine unrelated families diagnosed with IM revealed two disease-causing mutations in *PDGFRB*: c.1978C>A (p.P660T) and c.1681C>T (p.R561C) [1]. Interestingly, one family did not have either of these *PDGFRB* mutations, but all affected individuals had a c.4556T>C (p.L1519P) mutation in *NOTCH3*. The germline mutation c.1681C>T (p.R561C) in *PDGFRB* was also detected in 11 individuals with familial IM [7]. In addition, one individual harbored a c.1998C>A (p.N666K) somatic mutation. Very recently, a novel *PDGFRB* mutation (c.1679C>T; p.P560L) was identified in a 3-generation family with multicentric IM [11].

Platelet-derived growth factors (PDGFs) and PDGF receptors (PDGFRs) have important functions in the regulation of cell growth and survival [12]. The PDGF family consists of four structurally related single polypeptide units that constitute five functional homo- or heterodimers: PDGF-AA, PDGF-BB, PDGF-AB, PDGF-CC, and PDGF-DD [13]. PDGFs act via two receptor tyrosine kinases (RTKs), PDGFR-alpha and PDGFR-beta [14]. Both receptors can activate many major signal transduction pathways, including the Ras/MAPK, PI3K/Akt and phospholipase C-gamma pathways [15].

Moreover, other genes were associated with IM etiology, which demonstrates the possible genetic heterogeneity of this disease. As mentioned above, a connection between a c.4556T>C (p.L1519P) mutation in *NOTCH3* and IM was described in one study [1]. Human cells express four different Notch receptors, Notch 1–4, each encoded by a different gene [16]. The expression of *PDGFRB* can be regulated by Notch activity, as PDGFR-beta expression can be robustly upregulated by Notch 1 and Notch 3 signaling [17]. Another example is a c.511G>C (p.V171L) mutation in the potential tumor suppressor *NDRG4* that was associated with IM in one case [18]. In the same year, it was demonstrated that the c.1276G>A (p.V426M) mutation in *PTPRG* (protein tyrosine phosphatase, receptor type G) was able to substantially influence the penetrance of a c.1681C>T (p.R561C) mutation in *PDGFRB* [19]. *PTPRG* encodes an enzyme that could dephosphorylate PDGFR-beta and thus reduce PDGFR-beta activity [19,20].

A recent work revealed that two IM-associated mutations in *PDGFRB*, c.1681C>T (p.R561C) and c.1998C>A (p.N666K), constitutively activate PDGFR-beta and can induce cancer development in vivo [21]. The same study showed that cells harboring p.R561C and p.N666K mutations are sensitive to specific tyrosine kinase inhibitors, which were able to decrease PDGFR-beta phosphorylation and downstream signaling. These results suggested that blocking PDGFR-beta activity would offer a therapeutic option for IM treatment. Indeed, in a recently published study, targeted treatment with sunitinib and low-dose vinblastine led to a robust response in a child with refractory multiple IM and a c.1681C>T (p.R561C) mutation in *PDGFRB* [8].

In this work, we demonstrate for the first time the efficacy of sunitinib, erlotinib, U0126 and FR180204 on the cell line harboring a c.1681C>T (p.R561C) *PDGFRB* mutation found in patients with IM. Sunitinib is known as an inhibitor of several kinases, including PDGFR-beta [22], erlotinib is an inhibitor of epidermal growth factor receptor (EGFR) [23], U0126 inhibits MEK1/2 phosphorylation [24], and FR180204 inhibits ERK1/2 phosphorylation. These inhibitors were chosen on the basis of our previous findings [8] as well as on the results of subsequent phosphoprotein profiling of the NSTS-47 cell line.

## 2. Results

### 2.1. Germline Mutations in PDGFRB Were Identified in Both Children, and the Same Mutation in PDGFRB Was Confirmed in NSTS-47 Cells

Genetic analyses revealed that both siblings harbor a heterozygous germline c.1681C>T (p.R561C) mutation in the *PDGFRB* gene (Table 1). It was also confirmed that NSTS-47 cell line harbors the same heterozygous germline mutation c.1681C>T (p.R561C) in *PDGFRB*.

### 2.2. PDGFR-Beta, EGFR and ERK1/2 Kinases Are Highly Phosphorylated in Cells Harboring c.1681C>T (p.R561C) Mutation in PDGFRB

**Figure 1:**
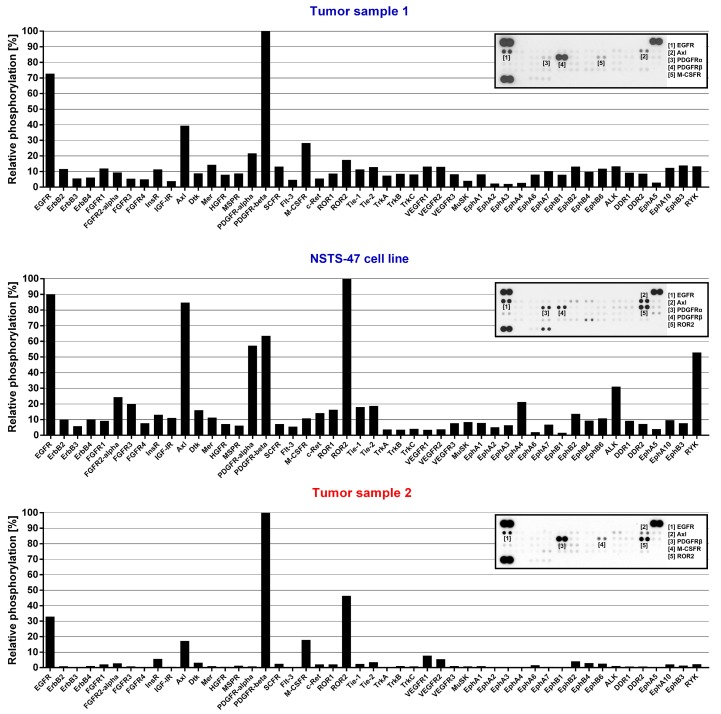
Phospho-receptor tyrosine kinases (RTK) array analysis. The relative phosphorylation of 49 RTKs was analyzed in tumor tissue obtained from the boy when he was 3.5 months old (Tumor sample 1), in the NSTS-47 cell line (derived from a tumor tissue of the boy obtained when he was 1 year and 7 months old) and in the tumor tissue of his 8-year-old sister (Tumor sample 2). platelet-derived growth factor receptor beta (PDGFR-beta) and epidermal growth factor receptor (EGFR) exhibited high levels of phosphorylation in all cases. Phosphorylation in NSTS-47 cells was measured after 24 h of serum-free cultivation. The array images captured using X-ray film are shown for each sample, and the five most phosphorylated receptor tyrosine kinases (RTKs) are marked. The upper part of the figure (Tumor sample 1) was already published in our previous case report [8] under the Creative Commons Attribution 4.0 International License.

**Figure 2:**
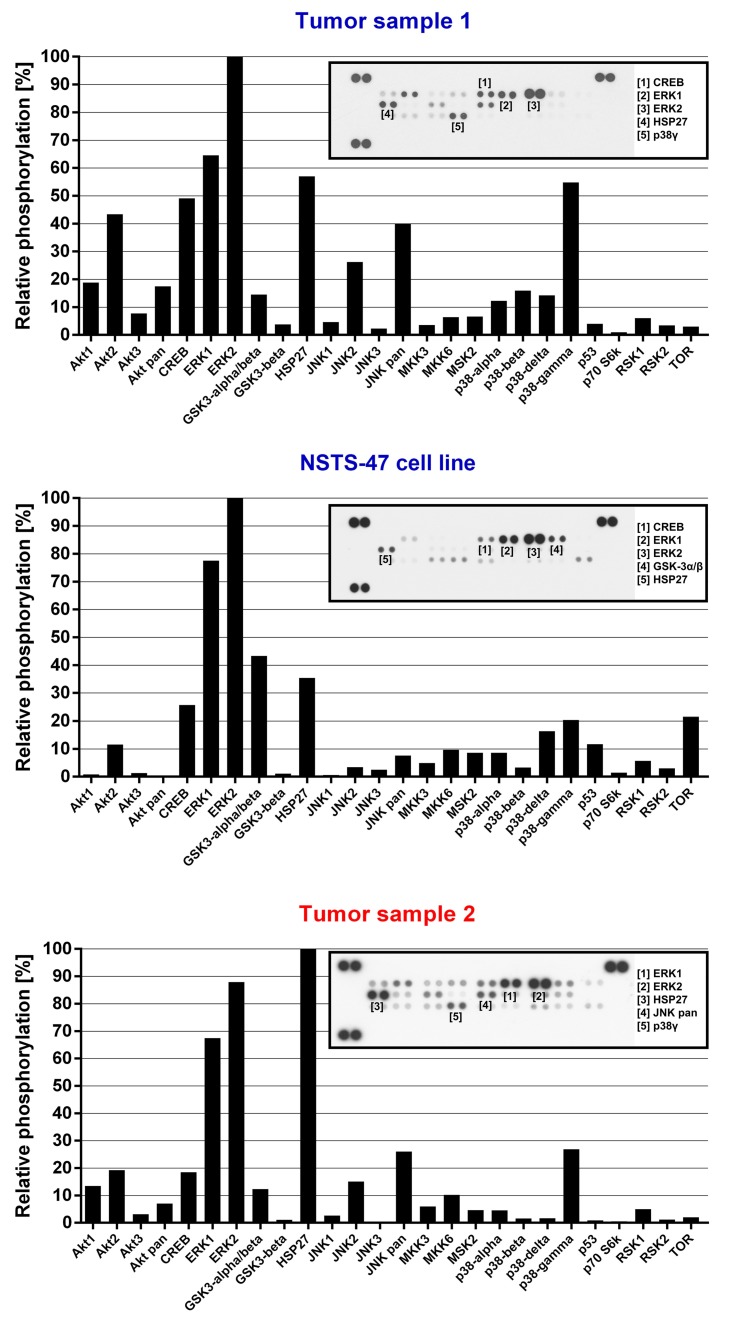
Phospho-mitogen-activated protein kinase (MAPK) array analysis. The relative phosphorylation of 26 signaling proteins, including 9 MAPKs, was detected in tumor tissue obtained from the boy when he was 3.5 months old (Tumor sample 1), in the NSTS-47 cell line (derived from a tumor tissue of the boy obtained when he was 1 year and 7 months old) and in the tumor tissue of his 8-year-old sister (Tumor sample 2). ERK1/2 exhibited high levels of phosphorylation in all cases. Phosphorylation levels in NSTS-47 cells was measured after 24 h of serum-free cultivation. The array images captured using X-ray film are shown for each sample, and the five most phosphorylated proteins are marked.

Given that both siblings and NSTS-47 cells harbor the c.1681C>T (p.R561C) mutation in *PDGFRB* and that PDGFR-beta c.1681C>T (p.R561C) mutants are constitutively phosphorylated and can activate various signaling pathways [21], we assessed the phosphorylation level of 49 RTKs and 26 other signaling proteins in tumor samples as well as in NSTS-47 cells. NSTS-47 cells were harvested, and phosphorylation levels were analyzed after cultivation for 24 h in Dulbecco’s modified Eagle’s medium (DMEM) without fetal calf serum (FCS) to eliminate the effects of various serum growth factors on the phosphorylation of the studied proteins. The screening of all 75 proteins showed that PDGFR-beta, EGFR (Figure 1) and ERK1/2 (Figure 2) kinases exhibited very high levels of phosphorylation in all samples. High levels of phosphorylation were also observed for ROR2, AXL (Figure 1), HSP27 and p38-gamma (Figure 2). These results confirmed that some kinases (namely, PDGFR-beta, EGFR and ERK1/2) were constitutively activated, as the high phosphorylation levels of these proteins were easily detectable in both tumor samples and in NSTS-47 cells after cultivation under serum-free conditions for 24 h.

### 2.3. NSTS-47 Cells Are Sensitive to Sunitinib and Erlotinib

It was confirmed that cells with the mutation c.1681C>T (p.R561C) in *PDGFRB* are sensitive to the tyrosine kinase inhibitors imatinib, nilotinib and ponatinib [21]. Given the phosphorylation profile in the NSTS-47 cell line, whether specific tyrosine kinase inhibitors could affect the proliferation of this cell line was assessed. NSTS-47 cells were first treated with sunitinib. Sunitinib was chosen for several reasons: (1) The NSTS-47 cell line harbors a c.1681C>T (p.R561C) mutation in *PDGFRB*, and PDGFR-beta was substantially phosphorylated in these cells; (2) sunitinib treatment inhibits PDGFR-beta phosphorylation [25]; and (3) sunitinib was successfully used to treat the boy with IM whose tumor tissue was used to generate the NSTS-47 cell line [8].

**Figure 3:**
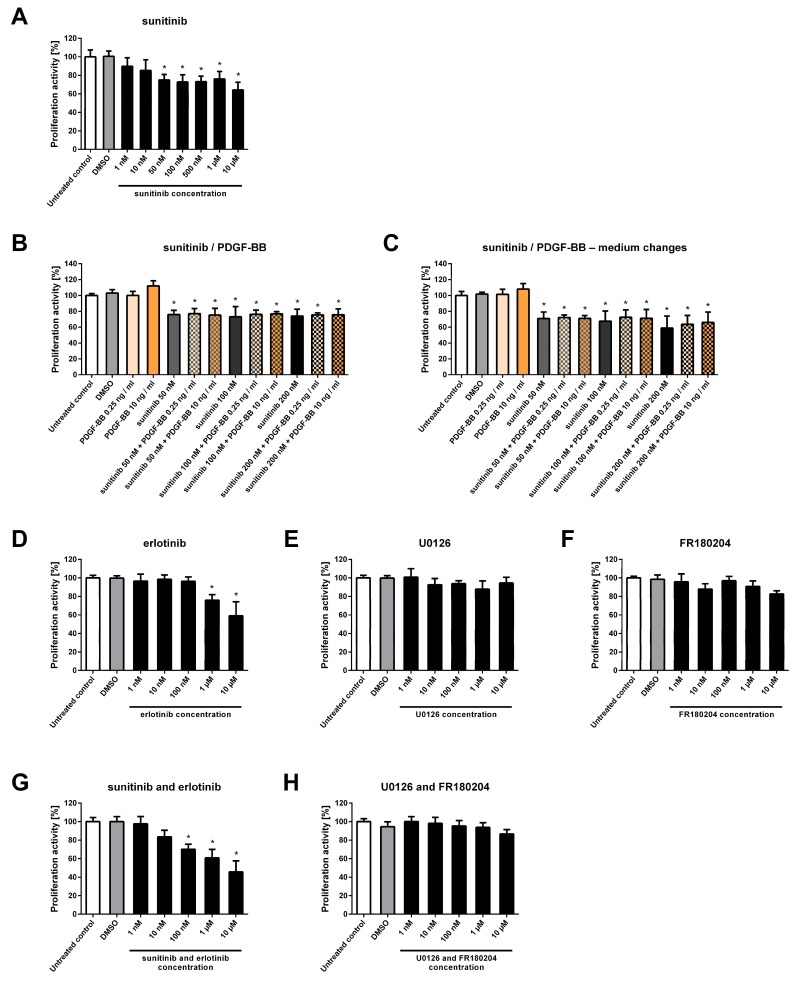
Proliferative activity of NSTS-47 cells after various experimental treatments. Proliferative activity was measured using an MTT assay after 6 days of incubation. The data represent the mean ± SD. Experiments were repeated three times in hexaplicate (**A**,**D**–**H**) or in triplicate (**B**,**C**). * *p* < 0.05 indicates a significant difference compared to control cells. (**A**) Sunitinib significantly decreased the proliferative activity of NSTS-47 cells. (**B**) NSTS-47 cells were sensitive to sunitinib, and this effect was not influenced by the presence of PDGF-BB at a high concentration (10 ng/mL). (**C**) Medium containing inhibitor and PDGF-BB was changed every 24 h during cultivation, which had no significant effect on the efficacy of the inhibitor. (**D**) NSTS-47 cells were also sensitive to erlotinib, as this inhibitor significantly affected cell proliferation. (**E**) No significant effect was observed after U0126 treatment. (**F**) FR180204 also did not significantly affect proliferative activity. (**G**) The combination of erlotinib and sunitinib significantly decreased the proliferative activity of NSTS-47 cells. (**H**) The combination of U0126 and FR180204 did not have a significant effect on NSTS-47 cell proliferation.

Cells were treated for six days with various concentrations of sunitinib, and after incubation, the proliferative activity was determined using the MTT assay. At sunitinib concentrations of 50 and 100 nM, which can be achieved in the plasma of children treated with sunitinib [26], the proliferative activity of NSTS-47 cells was significantly decreased (Figure 3A). In addition, 50 nM and 100 nM sunitinib decreased the proliferative activity of NSTS-47 cells to 75% and 73%, respectively, after six days.

To verify whether the observed effect of sunitinib is robust, NSTS-47 cells were cultivated with sunitinib in medium supplemented with PDGF-BB. A significant decrease in proliferative activity was observed after sunitinib treatment even when the cells grew in medium supplemented with PDGF-BB at a high concentration of 10 ng/mL (Figure 3B). In some experiments, the cultivation medium was changed every 24 h, and new medium with fresh inhibitor and fresh PDGF-BB was added (at medium changes) to prevent the potential degradation of sunitinib and PDGF-BB (Figure 3C).

Next, NSTS-47 cells were treated with erlotinib, U0126 and FR180204. These three inhibitors were chosen based on EGFR and ERK1/2 phosphorylation in NSTS-47 cells (Figure 1 and Figure 2). The ability of the combination of sunitinib and erlotinib to block both highly phosphorylated RTKs was tested, and a combination of U0126 and FR180204 was used to block the MEK/ERK signaling pathway.

At an erlotinib concentration of 1 µM, which can be achieved in the plasma of children treated with erlotinib [27], the proliferative activity of the NSTS-47 cell line was significantly decreased to 75% after 6 days of cultivation (Figure 3D). In contrast, NSTS-47 cells were not sensitive to U0126 and FR180204 because treatment of the NSTS-47 cell line with these inhibitors did not induce a significant decrease in proliferative activity (Figure 3E,F).

The combination of erlotinib and sunitinib also significantly decreased the proliferative activity of NSTS-47 cells (Figure 3G), but the effect of this combined treatment was similar to the effects of sunitinib or erlotinib alone. For instance, 100 nM sunitinib and 100 nM erlotinib decreased the proliferative activity to 70% (Figure 3G), but 100 nM sunitinib alone decreased the proliferative activity to 73% (Figure 3A). Another example is the combination of 1 µM erlotinib and 1 µM sunitinib; this treatment decreased the proliferative activity to 61% (Figure 3G), but 1 µM erlotinib alone decreased the proliferative activity to 75% (Figure 3D), and 1 µM sunitinib decreased the proliferative activity to 76% after six days (Figure 3A). Therefore, the combination of sunitinib and erlotinib did not have a significant additional effect on the reduction of NSTS-47 cell proliferation. In addition, the combination of U0126 and FR180204 did not show any significant effect on proliferative activity (Figure 3H).

Taken together, our results demonstrate that sunitinib and erlotinib can significantly decrease the proliferative activity of NSTS-47 cells, which harbor a c.1681C>T (p.R561C) mutation in *PDGFRB*, at concentrations that are achievable for these inhibitors in children plasma. However, the combination of sunitinib and erlotinib did not show an additional significant effect on cell proliferation. The inhibitors FR180204 and U0126 also did not have a significant effect on NSTS-47 cell proliferation.

### 2.4. PDGFR-Beta and EGFR Exhibited Ligand-Dependent Tyrosine Phosphorylation

**Figure 4:**
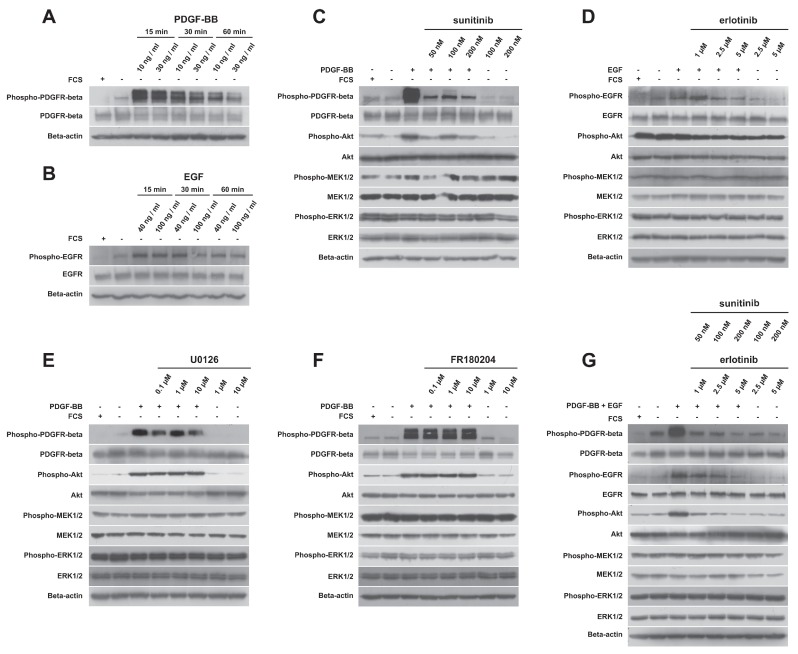
Analysis of protein phosphorylation. (**A**) PDGFR-beta phosphorylation is increased in response to PDGF-BB. Cells were stimulated for 15, 30 or 60 min using two different concentrations (10 ng/mL and 30 ng/mL) of PDGF-BB. (**B**) EGFR phosphorylation is increased in response to epidermal growth factor (EGF). Cells were stimulated for 15, 30 or 60 min using two different concentrations (40 ng/mL and 100 ng/mL) of EGF. (**C**) Sunitinib was able to decrease PDGFR-beta and Akt phosphorylation but not MEK1/2 and ERK1/2 phosphorylation. (**D**) Erlotinib decreased EGFR and Akt phosphorylation but had no effect on MEK1/2 and ERK1/2 phosphorylation. (**E**) U0126 treatment did not decrease MEK1/2 phosphorylation. (**F**) FR180204 treatment did not cause any changes in ERK1/2 phosphorylation. (**G**) The combination of sunitinib and erlotinib decreased PDGFR-beta, EGFR and Akt phosphorylation, but MEK1/2 and ERK1/2 phosphorylation was not affected.

Considering that only some kinase inhibitors significantly decreased the proliferative activity of the NSTS-47 cell line, detailed analyses of target kinases that should be affected by previously used inhibitors were performed using Western blotting. First, it was observed that the constitutively phosphorylated receptors PDGFR-beta and EGFR in NSTS-47 cells can respond to their ligands: Our results show that phosphorylation of both receptors was considerably increased in response to PDGF-BB or EGF (Figure 4A,B). Cell populations were serum starved for 24 h and then stimulated for 15, 30 or 60 min using two different concentrations of PDGF-BB or EGF. The cells that were serum starved for only 24 h and cells that were cultivated with FCS were used as negative controls. Receptor phosphorylation was significantly increased after 15 min, and then decreased in a time-dependent manner. Surprisingly, serum-starved cells that were not stimulated with PDGF-BB or EGF also exhibited an increase in receptor phosphorylation, in comparison to serum-cultivated cells. These experiments demonstrated that both receptors were functional and were able to activate downstream signaling molecules. 

### 2.5. Detailed Analysis of Signaling Pathways Revealed Constitutive Phosphorylation of MEK1/2 and ERK1/2 Proteins

In the next step, we analyzed the phosphorylation of PDGFR-beta, EGFR and downstream kinases, which can be activated by these RTKs after treatment with kinase inhibitors. In all experiments, cells were cultivated for 24 h in medium containing an inhibitor but not FCS. After 24 h, some cells were stimulated with PDGF-BB or/and EGF for 15 min to observe the effects of inhibitors on ligand-stimulated cells. Cells that were serum starved for only 24 h, and cells that were cultivated with FCS were used as negative controls.

Sunitinib alone decreased the phosphorylation of PDGFR-beta (Figure 4C). Akt phosphorylation was also decreased after sunitinib treatment, but a substantial decrease in MEK1/2 and ERK1/2 phosphorylation was not observed. Erlotinib decreased the phosphorylation of EGFR, but only at higher concentrations, and Akt phosphorylation was also slightly decreased (Figure 4D). No effect of erlotinib on MEK1/2 and ERK1/2 phosphorylation was observed. Surprisingly, U0126 did not decrease the phosphorylation of MEK1/2 (Figure 4E). Similarly, FR180204 treatment had no effect on ERK1/2 phosphorylation (Figure 4F). As expected, the combination of sunitinib and erlotinib markedly decreased the phosphorylation of PDGFR-beta, EGFR and Akt, but no effect was observed on MEK1/2 and ERK1/2 phosphorylation (Figure 4G).

Altogether, sunitinib and erlotinib showed inhibitory effects on RTKs and Akt. Interestingly, no substantial changes in MEK1/2 and ERK1/2 phosphorylation were observed after treatment with any inhibitor.

### 2.6. Serum Starvation of NSTS-47 Cells Induces an Increase in PDGFA Expression

**Figure 5:**
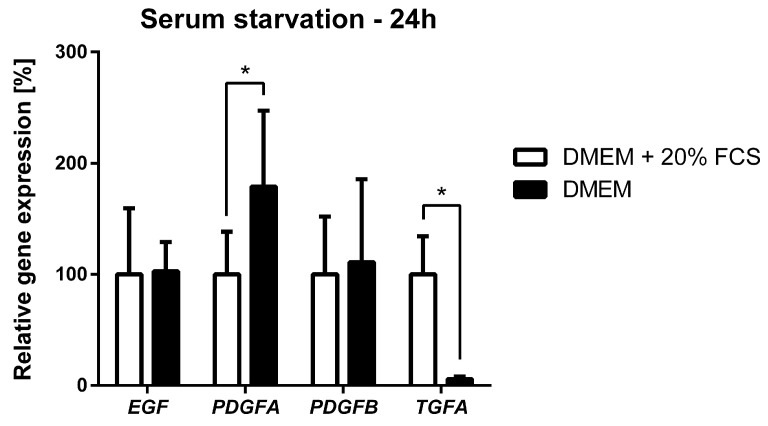
Effect of serum starvation on *EGF*, *PDGFA*, *PDGFB* and *TGFA* expression in the NSTS-47 cell line. Cells were cultivated in Dulbecco’s modified Eagle’s medium (DMEM) supplemented with 20% FCS or in DMEM without FCS. After 24 h, cells were harvested, and the expression of selected genes was analyzed using qPCR. The results represent the mean ± SD of nine (six in case of *PDGFB*) independent experiments. * *p* < 0.05 indicates statistically significant differences.

In some cases, our data indicated higher phosphorylation of PDGFR-beta and EGFR in serum-starved cells than in cells cultivated in DMEM supplemented with FCS (Figure 4A,B). Therefore, the expression of selected EGFR and PDGFR-beta ligands was measured to investigate whether there is a possible autocrine PDGF/PDGFR or EGF (TGF-alpha)/EGFR signaling loop that could contribute to the higher phosphorylation of RTKs. Expression of *EGF*, *PDGFA*, *PDGFB* and *TGFA* was analyzed under normal serum conditions (DMEM supplemented with 20% FCS) and under serum starvation conditions using qPCR. Substantial differences were observed in the transcriptional response of serum-starved cells (Figure 5). qPCR analyses also showed increased levels of *PDGFA* expression, while *EGF* and *PDGFB* mRNA levels were not significantly influenced by serum starvation, and *TGFA* expression was considerably decreased. 

## 3. Discussion

IM is a rare disorder of mesenchymal proliferation that is characterized by the development of nonmetastatic tumors [1]. Several studies have confirmed that specific point mutations in the *PDGFRB* gene are involved in the pathogenesis of IM [1,7,11]. However, mutations in the *PDGFRB* gene presumably show incomplete penetrance and variable expressivity, and other genes may be involved in the pathogenesis of IM [1,18,19].

The main goal of this study was to analyze the effects of various protein kinase inhibitors (PKIs) on the NSTS-47 cell line, which harbors the IM-associated c.1681C>T (p.R561C) mutation in *PDGFRB*. The results showed that sunitinib, a potent inhibitor of PDGFR-beta phosphorylation, can significantly decrease the proliferation of NSTS-47 cells.

Previously published results [21] show that PDGFR-beta p.R561C mutant cells have constitutively phosphorylated PDGFR-beta and are able to induce the phosphorylation of ERK1/2, PLC-gamma, STAT3, STAT5 and Akt in the absence of PDGF. These results are in accordance with our observations. We found that PDGFR-beta and ERK1/2 kinases were highly phosphorylated in both s and even in NSTS-47 cells that were serum starved for 24 h. We also detected increased phosphorylation of Akt2 in Tumor Sample 1.

The same study that revealed a role for the p.R561C mutation in PDGFR-beta [21] showed that imatinib, nilotinib, and ponatinib can decrease PDGFR-beta phosphorylation and inhibit cell proliferation. We studied the effects of sunitinib, a multi-tyrosine kinase inhibitor that is able to target PDGFR-beta. Sunitinib was chosen because siblings from whom tumor tissue samples were obtained responded very well to treatment with this inhibitor [8]. Sunitinib alone significantly decreased the proliferative activity of the NSTS-47 cell line, and this finding could explain the response of the siblings to the targeted therapy.

Western blot analyses showed that sunitinib is able to decrease the phosphorylation of mutant PDGFR-beta even in the presence of high PDGF-BB levels and can also decrease the phosphorylation of Akt. Because activated Akt is a well-established survival factor [28], these effects of sunitinib on PDGFR-beta and Akt phosphorylation can explain why sunitinib reduced the proliferative activity of NSTS-47 cells.

A similar inhibitory effect was observed for EGFR and erlotinib (the inhibitor of EGFR phosphorylation). Erlotinib also decreased NSTS-47 cell proliferation, and Western blot analysis showed that it was able to decrease EGFR and Akt phosphorylation. However, neither sunitinib nor erlotinib inhibited the phosphorylation of the corresponding receptor completely, and some receptor molecules remained phosphorylated even when high doses of those inhibitors were used.

Surprisingly, phosphorylation of MEK1/2 and ERK1/2 proteins was not significantly influenced by any inhibitor. This observation could explain why sunitinib and erlotinib incompletely decreased proliferative activity and why U0126 and FR180204 did not influence proliferative activity. MEK1/2 and ERK1/2 belong to the Ras/MAPK signaling cascade, which transmits signals from receptors and participate in regulating the cell cycle, apoptosis and differentiation [29]. All tyrosine kinase inhibitors have been previously shown to be able to simultaneously decrease PDGFR-beta and ERK1/2 phosphorylation, which resulted in the inhibition of proliferative activity [21]. In NSTS-47 cells, sunitinib and erlotinib decreased the phosphorylation of PDGFR-beta, EGFR and Akt, but for yet unknown reasons, MEK1/2 and ERK1/2 kinases remained phosphorylated at levels that were comparable with those detected in untreated cells.

Interestingly, incomplete penetrance of the c.1681C>T (p.R561C) mutation was found in a family with two children suffering from IM [19]. Genetic analyses revealed a c.1681C>T (p.R561C) mutation in *PDGFRB* in both siblings and, surprisingly, also in their healthy mother. However, both siblings had inherited a heterozygous c.1276G>A (p.V426M) mutation in *PTPRG* from their healthy father. The *PTPRG* gene encodes a protein called receptor-type tyrosine-protein phosphatase gamma that can dephosphorylate PDGFR-beta [19,20]. Therefore, the mutation in *PTPRG* could probably decrease the efficiency of the phosphatase to dephosphorylate its substrates and thus positively influence the phosphorylation of PDGFR-beta and the penetrance of mutant *PDGFRB* [19].

Finally, our analyses of gene expression showed that the phosphorylation status of PDGFRs in NSTS-47 cells was not influenced by only mutations in PDGFR-beta. We analyzed the gene expression levels of *EGF*, *PDGFA*, *PDGFB* and *TGFA* in NSTS-47 cells that were serum starved for 24 h. The expression of *TGFA* decreased, but no difference was observed in the expression of *EGF* and *PDGFB*; however, *PDGFA* gene expression was significantly increased. The increase in *PDGFA* expression was unexpected and could result in the stimulation of cells via an autocrine mechanism, an increase in PDGFR-alpha phosphorylation and improved survival of NSTS-47 cells in the absence of serum.

## 4. Materials and Methods

### 4.1. Tumor Samples

Two tumor samples and one tumor-derived cell line were used in this study. Tumor Sample 1 was obtained from a 3.5-month-old infant boy suffering from inborn generalized IM, and Tumor Sample 2 was obtained from his 8-year-old sister who was suffering from a skull base tumor and had a history of spontaneous regression of subcutaneous lesions. The Research Ethics Committee of the School of Medicine (Masaryk University, Brno, Czech Republic) approved the study protocol, and written informed consent was obtained from legal guardians of the siblings. A case report concerning these siblings was published recently [8].

### 4.2. Cell Line and Cell Culture

The NSTS-47 cell line was established in our laboratory with the procedure previously described [30]. A tumor sample was obtained from the same boy mentioned in the previous paragraph during curative surgical procedure when he was 1 year and 7 months old. Cells were grown in Dulbecco’s modified Eagle’s medium (DMEM), supplemented with 20% fetal calf serum (FCS), 2 mM glutamine, 100 IU/mL penicillin and 100 µg/mL streptomycin (all purchased from GE Healthcare Europe GmbH, Freiburg, Germany). The cell line was maintained under standard conditions at 37 °C in a humidified atmosphere containing 5% CO_2_ and subcultured one or two times per week. Cells from passage number 8 to 19 were used for experiments.

### 4.3. Genetic Analyses

The mutation in *PDGFRB* was identified by Sanger sequencing using an ABI 3130 Genetic Analyzer (Applied Biosystems, Foster City, CA, USA) and confirmed by whole exome sequencing (WES). In all cases, WES was performed using the TruSeq Exome Kit, NextSeq^®^ 500/550 Mid Output Kit v2 and NextSeq 500 (all Illumina, San Diego, CA, USA). 

### 4.4. Chemicals

Sunitinib, erlotinib, U0126 (all purchased from Cell Signaling Technology, Danvers, MA, USA) and FR180204 (Sigma-Aldrich, St. Louis, MO, USA) were prepared as a 20 mM stock solution in dimethyl sulfoxide (DMSO) and stored at −20 °C. PDGF-BB (Cell Signaling Technology) was prepared at a concentration of 100 µg/mL in 20 mM citric acid (pH 3.0) supplemented with 0.8% BSA (bovine serum albumin) and stored at 4 °C. EGF (Sigma-Aldrich) was prepared at a concentration of 100 µg/mL in 10 mM HCl and stored at 4 °C. For the determination of proliferative activity, concentrations of protein kinase inhibitors (PKIs) ranging from 0.001 to 10 μM and PDGF-BB concentrations of 0.25 and 10 ng/mL were tested. For Western blot analyses, PKI concentrations ranging from 0.05 to 10 μM, PDGF-BB concentrations of 10 and 30 ng/mL and EGF concentrations of 40 and 100 ng/mL were used.

### 4.5. Phospho-RTK and Phospho-MAPK Array Analysis

The relative phosphorylation levels of 49 RTKs were analyzed using the Human Phospho-RTK Array kit (R&D Systems, Minneapolis, MN, USA), and the relative phosphorylation levels of 26 proteins, including 9 MAPKs, were determined using the Human Phospho-MAPK Array kit (R&D Systems) according to the manufacturer’s protocol. The levels of phosphorylation were quantified using ImageJ software [31] and normalized to control spots and the background. The analysis was performed as described in previous studies [8,32].

### 4.6. MTT Assay

The MTT assay was used to determine the proliferative activity of the NSTS-47 cell line. A total of 10^3^ cells were seeded in 200 μL of culture medium into each well of 96-well microplates, and cells were allowed to adhere overnight. The next day, the medium was carefully removed, and fresh medium containing various concentrations of chemicals described above or control medium was added. The microplates were incubated under standard conditions. To evaluate changes in cell proliferation, the medium was removed and replaced with 200 μL of fresh DMEM containing 3-(4-dimethylthiazol-2-yl)-2,5-diphenyltetrazolium bromide (MTT) at a concentration of 0.5 mg per mL. The microplates were then incubated at 37 °C for 3.5 h. The medium was carefully removed, and the formazan crystals were dissolved in 200 μL of DMSO. The absorbance was measured at 570 nm using a Sunrise Absorbance Reader (Tecan, Männedorf, Switzerland), with a reference absorbance at 620 nm.

### 4.7. Western Blotting and Immunodetection

Whole-cell extracts were loaded onto 10% polyacrylamide gels, electrophoresed, and blotted on polyvinylidene difluoride membranes (Bio-Rad Laboratories, Munich, Germany). The membranes were blocked with 5% nonfat dry milk in phosphate buffered saline (PBS) containing 0.1% Tween-20 and incubated overnight with the corresponding primary antibody. The primary and secondary antibodies used in this study are shown in Table 2. Membranes were incubated with corresponding secondary antibodies for 1 h. ECL-Plus detection was performed according to the manufacturer’s instructions (GE Healthcare, Little Chalfont, UK).

### 4.8. RT-qPCR

The relative expression levels of selected genes were studied using RT-qPCR. Total RNA was extracted using the GenElute™ Mammalian Total RNA Miniprep kit (Sigma-Aldrich), and RNA concentration and purity were determined spectrophotometrically. For all samples, equal amounts of RNA were reverse transcribed into cDNA using M-MLV reverse transcriptase (Top-Bio, Prague, Czech Republic). RT-qPCR was carried out in 10 μL reaction volumes using the KAPA SYBR^®^ FAST qPCR Kit (Kapa Biosystems, Wilmington, MA, USA) and analyzed using the 7500 Fast Real-Time PCR System and 7500 Software v. 2.0.6 (both Life Technologies, Carlsbad, CA, USA). Changes in the transcript levels were determined using the 2^−ΔΔ*C*T^ method [33]. The housekeeping gene *HSP90AB1* was used as an endogenous reference control. The primers used in this study are listed in Table 3.

### 4.9. Statistical Analysis

Quantitative data are shown as the mean ± standard deviation (SD). Data from MTT assays were analyzed using one-way ANOVA followed by Dunnett’s test; *p* < 0.05 was considered statistically significant. The qPCR data were analyzed using the Mann-Whitney test (two-tailed); *p* < 0.05 was considered statistically significant.

## 5. Conclusions

To conclude, our work demonstrated that tumor cells with the c.1681C>T (p.R561C) mutation in *PDGFRB* show high levels of PDGFR-beta and ERK1/2 phosphorylation. Furthermore, our data support the use of specific tyrosine kinase inhibitors targeting PDGFR-beta phosphorylation as a treatment suitable for IM. This is the first study to show that sunitinib is able to reduce the proliferative activity of IM cells with a c.1681C>T (p.R561C) mutation in vitro.

## Figures and Tables

**Table 1:** Germline mutations identified in patients.

Gender	Age	*PDGFRB* Mutation
Male	3.5 months	c.1681C>T (p.R561C)
Female	8 years	c.1681C>T (p.R561C)

**Table 2:** Primary and secondary antibodies.

**Primary Antibodies**				
Antigen		Manufacturer	Catalog No.	Dilution
Beta-actin		Sigma-Aldrich	A5441	1:20,000
Akt (pan)		Cell Signaling Technology	4691	1:1000
Phospho-Akt (Ser473)		Cell Signaling Technology	4060	1:2000
ERK1/2		Cell Signaling Technology	4695	1:1000
Phospho-ERK1/2 (Thr202/Tyr204)		Cell Signaling Technology	4370	1:2000
MEK1/2		Cell Signaling Technology	9122	1:1000
Phospho-MEK1/2 (Ser217/221)		Cell Signaling Technology	9121	1:1000
EGFR		Cell Signaling Technology	2646	1:1000
Phospho-EGFR (Tyr1068)		Cell Signaling Technology	2236	1:1000
PDGFR-beta		Cell Signaling Technology	3169	1:1000
Phospho-PDGFR-beta (Tyr751)		Cell Signaling Technology	4549	1:1000
**Secondary antibodies**				
Specificity	Conjugate	Manufacturer	Catalog No.	Dilution
Anti-Mouse IgG	horseradish peroxidase	Cell Signaling Technology	7076	1:2000–1:20,000
Anti-Rabbit IgG	horseradish peroxidase	Cell Signaling Technology	7074	1:2000

**Table 3:** Primers.

Gene	Gene Symbol	Primer Sequence
Epidermal growth factor	*EGF*	F: 5′-AGGATTGACACAGAAGGAACCAA-3′ R: 5′-ACATACTCTCTCTTGCCTTGACC-3′
Heat shock protein 90 alpha family class B member 1	*HSP90AB1*	F: 5′-CGCATGAAGGAGACACAGAA-3′ R: 5′-TCCCATCAAATTCCTTGAGC-3′
Platelet derived growth factor subunit A	*PDGFA*	F: 5′-TCCGTAGGGAGTGAGGATTCTTT-3′ R: 5′-GGCTTCTTCCTGACGTATTCCA-3′
Platelet derived growth factor subunit B	*PDGFB*	F: 5′-GATCCGCTCCTTTGATGATCTCC-3′ R: 5′-ATCTCGATCTTTCTCACCTGGAC-3′
Transforming growth factor alpha	*TGFA*	F: 5′-TGCCACTCAGAAACAGTGGTC-3′ R: 5′-AGTCCGTCTCTTTGCAGTTCTT-3′

F, forward primer; R, reverse primer.

## Abbreviations

DMEM	Dulbecco’s modified Eagle’s medium
DMSO	dimethyl sulfoxide
EGFR	epidermal growth factor receptor
ERK	extracellular signal-regulated kinase
FCS	fetal calf serum
IM	infantile myofibromatosis
MAPK	mitogen-activated protein kinase
MEK	MAPK/ERK kinase
MTT	3-(4-dimethylthiazol-2-yl)-2,5-diphenyltetrazolium bromide
PDGFR	platelet-derived growth factor receptor
PKIs	protein kinase inhibitors
RTKs	receptor tyrosine kinases
TGFA	transforming growth factor alpha
WES	whole exome sequencing
